# A Method of Data Aggregation for Wearable Sensor Systems

**DOI:** 10.3390/s16070954

**Published:** 2016-06-23

**Authors:** Bo Shen, Jun-Song Fu

**Affiliations:** School of Electronic and Information Engineering, Key Laboratory of Communication and Information Systems, Beijing Municipal Commission of Education, Beijing Jiaotong University, Beijing 100044, China; 12120067@bjtu.edu.cn

**Keywords:** wearable sensor systems, data query, routing tree, data aggregation

## Abstract

Data aggregation has been considered as an effective way to decrease the data to be transferred in sensor networks. Particularly for wearable sensor systems, smaller battery has less energy, which makes energy conservation in data transmission more important. Nevertheless, wearable sensor systems usually have features like frequently dynamic changes of topologies and data over a large range, of which current aggregating methods can’t adapt to the demand. In this paper, we study the system composed of many wearable devices with sensors, such as the network of a tactical unit, and introduce an energy consumption-balanced method of data aggregation, named LDA-RT. In the proposed method, we develop a query algorithm based on the idea of ‘happened-before’ to construct a dynamic and energy-balancing routing tree. We also present a distributed data aggregating and sorting algorithm to execute top-*k* query and decrease the data that must be transferred among wearable devices. Combining these algorithms, LDA-RT tries to balance the energy consumptions for prolonging the lifetime of wearable sensor systems. Results of evaluation indicate that LDA-RT performs well in constructing routing trees and energy balances. It also outperforms the filter-based top-*k* monitoring approach in energy consumption, load balance, and the network’s lifetime, especially for highly dynamic data sources.

## 1. Introduction

Wearable sensor systems consist of devices with one or many sensor nodes. Each sensor node is usually equipped with a low-speed microprocessor, limited memory, and a radio transceiver and receiver [1,2,3]. In real world, these wearable sensor systems usually form a wireless sensor network to transfer collected data. With the development of technologies, wearable sensor systems are used extensively in civilian and military fields, such as monitoring the environment and habitats, tracking objects, transferring data within a tactical unit in battlefield, detecting fires, and controlling traffic. Since the data generated in thus system is very large and dense, it is difficult to determine how to collect data in different environments. In many cases, people have only focused on a part of observed objects (for instance, the maximum or minimum values). Therefore, how to process a query and collect needed data in wearable sensor systems has become the subject of extensive and in-depth research and learning [4,5,6,7,8,9,10]. In general, the capabilities of communication, computing power, storage capacities, and battery lifetime of wearable devices are very limited. So, power consumption is still a major issue in wearable sensor systems and wireless sensor networks [11,12,13,14,15,16].

Unlike the traditional sense of sensor systems, wearable sensor systems usually form networks with dynamic topology and generate data over a large range. Clearly, the existing data-window filter and tree-based routing path methods can’t achieve good performance in these networks. First, tree-based routing algorithms, such as the directed diffusion algorithm, use sensor node’s level or distance from base station as the metric to construct data transmission path. Although the shortest path tree can be achieved, a relatively fixed path still leads to imbalances in energy consumption and is not suitable for networks with dynamic topology. Second, window-based algorithms also result in additional communication overhead when the filter is updating, especially when the measured data of sensor nodes change frequently over a large range. This is just the general circumstance in wearable sensor systems.

Regarding these issues, we introduce a novel top-*k* query paradigm. Unlike directed diffusion scheme, the proposed method utilizes the ‘happened-before’ relation of received messages, which is a concept used in distributed system and means one event happening before another [17], to determine which paths should be used, and constructs, on demand, a dynamic routing tree that considers the remaining energy, processing load, and the time drift of sensor nodes to avoid overconsumption of energy. This is specially designed for dynamic topology of wearable sensor systems. In order to reduce in-network traffic, each sensor node executes a data aggregation algorithm to merge the data measured by itself and transmitted from its children. Then, the base station extracts the top-*k* result after collecting all the messages. The data aggregation algorithm is a distributed sorting and query scheme that restricts the data-merging process inside sensor nodes and makes it possible for every node to send only one message during data-reporting procedure. Though wearable sensor systems are the considered network environment, the proposed methods could also be used in other wireless sensor networks.

We implement the proposed methods and algorithms over data link layer directly in the network simulation environment and test the performance from various aspects. We also compare the proposed method with filter and tree-based top-*k* monitoring methods with respect to the lifetime of network and the balance of energy consumption. The obtained results indicate that the proposed method produces a better balance of energy consumption and longer lifetime of network when there are large changes in the range of data.

The remainder of this paper is organized as follows: Section 2 discusses the related work on data aggregation and top-*k* query methods in wireless sensors networks. Section 3 presents our data aggregation and top-*k* query method in details. Then, we propose an approach for constructing routing tree. In Section 4, we evaluate the proposed method through extensive of simulations. Our conclusions and recommendations for further work are presented in Section 5.

## 2. Related Work

Generally, most of the energy is consumed by communication between nodes in wireless sensor networks, such as wearable sensor systems [11,14,18]. Therefore, minimizing the amount of traffic in processing queries can improve the efficiency of search and save a significant amount of energy, thereby prolonging the life of networks. Obviously, energy can be saved if the amount of data requested during a query could be minimized. Such query is called top-*k* query [5], which is considered as a kind of data aggregation methods. Top-*k* query is designed to determine the *k* highest (or lowest) observed values and is a very important task in data acquisition.

The pioneering work related to top-*k* query is deemed to be TAG (Tiny AGgregation) [6], despite the facts that the concept of top-*k* is not mentioned distinctly in it and the data grouping method proposed by TAG is different from top-*k*. TAG uses a tree-based routing scheme to construct its data aggregation data path, which is similar to directed diffusion [4]. In TAG, an in-network aggregation technique called ‘grouping’ was presented to reduce network traffic. When a query is pushing down through an established routing tree, an expression over one or more attributes is brought to every sensor node. According to the expression, the reading of each sensor is placed into one group, the groups are partitioned, and the values are aggregated in the appropriate groups when answers to the query are flowing back. The result is that the base station can collect aggregated data directly, and the data that each sensor node sends are reduced. However, data collecting and aggregated data controlling in TAG depend on time epoch, which covers all the levels of the tree. So, the depth of TAG tree is limited. Otherwise, the time epoch of one query round would be too long. In addition, sensor nodes near the tree’s root (usually base station) will consume more energy than other nodes [19], which leads to an imbalance in energy consumption.

Basing on TAG routing tree, Wu et al. proposed a data query method called the Filter-Based Monitoring Approach (FILA) [5]. Differing from the way of grouping data in TAG, FILA introduced a data filter to each sensor node to decrease unnecessary updates. The basic assumption is that readings of all sensor nodes will fall into a certain range, and, in most cases, the reading of a sensor node will change only in a small interval. Thus, the range of values could be divided into several intervals by a carefully planned schema, namely windows. For the nodes whose readings belong to top-*k* result set, non-overlapping, distinct windows should be assigned to each of them. Conversely, other nodes could share the same windows [5]. During the initial phase of data collection, the base station queries all readings from sensor nodes and sorts them to determine the top-*k* result set. Then, the base station calculates a filter window for each sensor node and sends it all nodes. At the following data report, only the nodes whose readings change beyond the filtering window should transmit their data to the base station. Then, the base station will re-calculate the filter settings. Apparently, the mechanism of filtering window will reduce the amount of data transmitted markedly when the readings of most sensor nodes in top-*k* result set do not go beyond their windows. Otherwise, sensor nodes will consume additional energy to update their filters.

Myungho Yeo et al. proposed a priority-based approach called PRIM [20]. Their basic idea was self-sorting by the value of sensor readings for collecting readings in an orderly manner. That is, sensor nodes must determine their sequence without any centralized controls. So, the authors proposed a data-aware, priority-assignment algorithm (DPA), which took advantage of the time slots in TDMA frame. In PRIM, a routing tree is established using hierarchical clustering and routing algorithms, such as LEACH [21] and HEED [22]. Based on similar thought, the authors also introduced a sequence-aware, top-*k* (SAT) monitoring scheme that also makes sensor nodes determine their order for the data-gathering phase [23].

Hang et al. studied filter-based, top-*k* queries and proposed three improvements, i.e., distributed top-*k* queries, setting filter values, and predicting the available interval for each node by ARMA [24]. EXTOK [3] is another top-*k* query solution, and it considers both tree topology and filtering-based query. Unlike other top-*k* query methods, EXTOK addresses the case in which the exact set of top-*k* values must be retrieved, regardless of how many nodes reported it. Zhang et al. [25] studied multi-dimensional, top-*k* query and used data-prediction method to establish the bi-boundary filter rule to refine data. Silberstein et al. [26] proposed to use samples of past sensor node readings to formulate the problem of optimizing approximate top-*k* queries under an energy constraint as a linear program. Threshold Join Algorithm (TJA) [27] is an efficient, top-*k* query algorithm that uses a non-uniform threshold on queried reading to minimize the redundancy of data. Babcock et al. studied the distributed, top-*k* monitoring problem with a user-specified error tolerance [28]. In their approach, first, an initial top-*k* set was computed. Then, the monitored nodes were installed with arithmetic constraints, which were used to ensure the continuing accuracy of the initial top-*k* set to within the user-supplied error tolerance. When a constraint is violated, it is re-computed by the coordinator (root node). Obviously, it is just an earlier version of filter-based, top-*k* query method.

Inevitably, top-*k* query must be based on the route of wireless sensor network. So, another key point is to determine how to construct an efficient route that will satisfy energy conservation demand of top-*k* query.

TAG uses a tree-based routing approach. The base station first broadcasts a message to build the tree of path. Nodes that receive this message will become the children of sender and will forward the message to their neighbors. This process is repeated until every node has received the message. After message broadcast, a tree of path will be rooted at the base station. Normally, it is a shortest-path-tree. As mentioned above, in such a structure, nodes close to tree’s root will consume more energy and shorten network’s lifetime. Further, in most cases when a tree is constructed, it will have the same structure as the preceding trees.

Directed diffusion (DD) is a data-centric dissemination protocol, i.e., all communication in DD is for named data. This makes it possible to save energy by selecting good paths [4]. The schematic for DD has three steps, i.e., (a) interest propagation; (b) setting up of the initial gradients, and (c) delivery of data along a reinforced path. These steps indicate that it is possible to deliver the named data through high-quality paths, but this requires an initial query flooded to explore paths [29]. MADD [29] applies DD in a multi-hop environment and uses the gradient of DD to dispatch the mobile agent (MA) [30]. The performance of MADD in the proposed gradient-based routing scheme was better than DD in terms of energy consumption.

Sensor Protocols for Information via Negotiation (SPIN) [31] is a family of data dissemination protocols for wireless sensor networks, including SPIN-PP, SPIN-BC, SPIN-RL, and SPIN-EC. The protocols use meta-data negotiation to eliminate the transmission of redundant data throughout a network. These negotiations ensure that nodes transmit data only when it is necessary, so they never waste energy on useless transmissions. Also, the protocols use resource-adaptation to distribute data efficiently when energy supply is limited. Because nodes are resource-aware, they reduce their activities when their resources are low to increase their longevity. SPIN-PP is used in networks for point-to-point communication media, and SPIN-BC is used in broadcast communication media. SPIN-EC and SPIN-RL are modified versions of the first two protocols.

The Low Energy Adaptive Clustering Hierarchy (LEACH) [21] is representative of the clustering structure, which partitions the sensor nodes into several clusters according to their positions in network. During operation, LEACH reconstructs the clusters in a circle. Each process of reconstructing the clusters is described as a ‘round’, and each round begins with a set-up phase during which the clusters are organized, and this is followed by a steady-state phase in which the data are transferred from the nodes to the head of cluster and to the base station. The cluster-building process has four stages, i.e., selecting the head of cluster, broadcasting the selection result establishing the cluster, and generating the scheduling mechanism. After clustering, member nodes select and join a suitable cluster head. In the steady-state phase, each member node sends the data it senses to its cluster head. The cluster head collects and aggregates the incoming data from its member node and sends them to the base station. Apparently, LEACH can be used to generate the path of top-*k* query. It can also be combined with data aggregation algorithm to reduce the amount of data to be transmitted.

Except the above common methods, there are some new ones in literature. Mo et al. combined network clustering with top-*k* query and introduced cluster-based routing for top-*k* querying (CRTQ) [19]. Ref. [32] proposed a tree structure named Partial Ordered Tree (POT) to maintain the clusters with highest readings. In POT, each node chooses a node with highest reading as its parent, and the highest node in a local area will act as the root of POT of this area. Then, a network is partitioned into many POTs, each of which has a root node. Essentially, POT is a routing tree construction method based on network clustering. It builds routing tree dynamically. The weakness of POT is that when a network has uniform readings, its performance declines evidently.

EXTOK [3] employed DST (Dominating Set Tree) [33] to construct routing tree and disseminate the query to all nodes. Differing from dissemination by flooding in SPT (Shortest Path Tree), the idea of DST is to ensure that ‘only the smallest possible subset of non-leaf nodes transmit the query’. DST connects nodes named dominating nodes [34] to a tree rooted at base station. Obviously, once a DST is constructed, the number of disseminated queries will reduce evidently. But finding dominating nodes is an NP-hard problem, though ref. [34] proposed a distributed method. Due to the complexity of DST construction, frequent reconstruction should be avoided. This results in a fixed routing tree used by EXTOK.

DAS (Data Aggregation Scheduling) [35] introduced a data aggregation scheduling based on maximal independent sets. It consists of two phases: constructing a distributed aggregation tree and performing distributed aggregation scheduling. In the first phase, connected dominating set is employed, which is also the basis of DST. So, DAS faces the same problems as DST.

Many researchers have shown that the method of route used in top-k query is closely related to the performance of query [3,5,19,23,24]. The same applies to the method of collecting data [7,15,35].

## 3. Local Data Aggregation Basing on Routing Tree

### 3.1. Problem

Consider a wireless sensor network composed of densely deployed wearable sensor devices, each of which monitors surrounding environment and measures physical phenomena (e.g., humidity, temperature, and residual energy) at a fixed rate determined by data gatherer. Suppose there is also a base station set up for serving as the bridge between the network and data gatherer and energy is supplied continuously to the base station. But, the resources (e.g., energy, communication band) of wearable devices are strictly limited. We then focus on how sensor nodes in the network respond to a top-*k* query of base station in an energy-efficient way.

Let $S=\left\{s_{i}:i=1,2,\ldots ,m\right\}$ be the sensor node set and $R=\left\{r_{i}:i=1,2,\ldots ,m\right\}$ be the corresponding reading value set of all nodes. For convenience, here we discuss maximum problem only. The task of top-*k* query can be expressed in the form,

(1) $$R^{*}=\underset{S^{*}}{\text{argmax}}\sum^{\text{​}}r_{q}\;\text{subject to}\;r_{q}\in R,\;s_{q}\in S^{*}\;\text{and}\;\left|R^{*}\right|=\left|S^{*}\right|=k$$

As discussed in Section 2, the aim of top-*k* query is to reduce energy consumption used in data transmission. However, it is challenging to determine how to find *k* highest readings from all sensor nodes distributed in a network. In addition to the filter-based method, distributed local data aggregation is a feasible idea. So, the issue is to make a way to achieve this kind of aggregation. In order to reach this goal, we propose a top-k query approach basing on routing tree, which includes two parts, local data aggregation and routing tree construction, as presented below.

### 3.2. Local Data Aggregation

**Figure 1:**
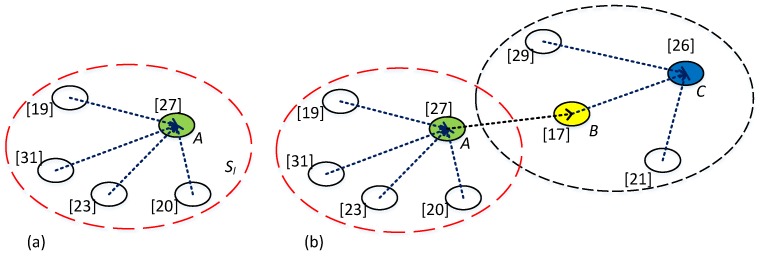
Kernel node and structure of LDA. (**a**) Single sub-network; (**b**) Cascaded sub-networks.

Suppose $S_{l}$ is a sub-network of sensor network S. We call $s^{\kappa }$ the kernel node of $S_{l}$ if

(2) $$\forall \;s_{i}\in S_{l},\;d\left(s_{i},s^{\kappa }\right)\leq \delta ,\;\text{and}\;d\left(s^{\kappa },s^{\tau }\right)\leq \delta \;\text{subject to}\;s^{\kappa }\in S_{l},\;S_{l}\subseteq S$$

where $s^{\tau }$ is the sink node or a node in another sub-network, $d\left(s_{1},s_{2}\right)$ is the physical distance between node $s_{1}$ and node $s_{2}$, and δ is the distance threshold of communication between two sensor nodes. From another perspective, if a sub-network $S_{l}$ satisfies Equation (2), $s^{\kappa }$ will be considered as the kernel node of $S_{l}$, and all other nodes in $S_{l}$, named member node, can communicate with it. For example, in Figure 1a,b, Node A is a kernel node with 4 member nodes. In Figure 1b Node C is also a kernel node with 3 member nodes. If Node A and Node B are considered a sub-network, Node B is the kernel and Node A is the member node.

**Figure 2:**
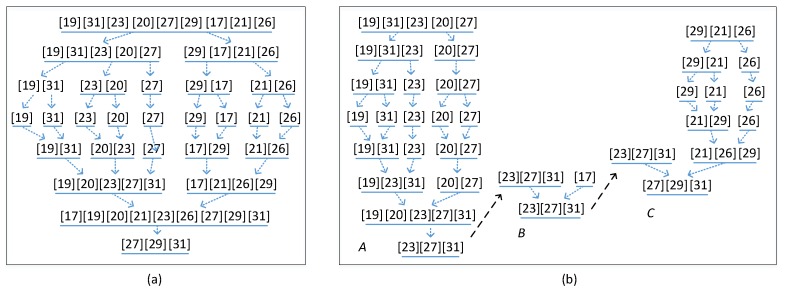
Example of LDA algorithm. (**a**) Merge sort algorithm; (**b**) Distributed merge sort algorithm.

Kernel node is charged with collecting all readings from its member nodes and itself and getting $R_{l}^{*},\;|R_{l}^{*}|\leq k$ defined by Equation (1), and then send $R_{l}^{*}$ to the sink node or a node in another sub-network, as shown in Figure 1b. We name this way Local Data Aggregation (LDA), in which kernel node employs a variant merge sort algorithm to obtain top *k* readings. The basic merge sort algorithm is as illustrated in Figure 2a. Figure 2b shows an example of LDA. In the example, Node A is the kernel node of a sub-network illustrated by red dashes circle. When Node A obtains all readings of the sub-network, it will calculate local top *k* by merge sort algorithm. And then Node A sends these *k* readings to its parent Node B which is the kernel node of another sub-network. Comparing with the whole network, $S_{l}$ has relatively lesser nodes obviously. In addition, a kernel node, such as Node C in Figure 2b, will receive no more than *k* readings from any of other kernel nodes. So, less data need to be sorted. Because kernel node uploads at most k sorted readings rather than all that it received, the traffic in network and sorting computations can be significantly reduced, e.g., Node C in Figure 2b. Further, unlike uploading message individually, up to *k* readings can be uploaded within one message, which means that every node must send only one message to the kernel node it connects to in a query round. It is important to balance the energy consumption of all sensor nodes in a network.

The complexities of space and calculation of LDA algorithm can be reduced further when messages are processed one by one as opposed to processing them all at the same time. In the worst case, *k* comparisons are made to aggregate a new reading. Because the stored list of values is in an ordered state at all times, the worst case is rare.

Obviously, the aggregation process is similar to the last merge step of the merge-sort algorithm [36]. So, its time complexity and space complexity are all O(*k*). If a kernel node has *m* child nodes, the time complexity will be O(*mk*) in the worst case performance, and the space complexity will still be O(*k*). LDA has the following characteristics, (1) distribute the sorting tasks into multiple nodes, which is beneficial for the balance of energy consumption; (2) restrict the maximal number of data in a message to *k*; (3) simplify the calculation of each node.

In most applications of wireless sensor networks, the parameter *k* is small, and kernel node has only a few child nodes. So, the values of the readings can be aggregated quickly and efficiently through the LDA approach proposed in this section. Further, local aggregation of LDA allows most data to be sent only once and installs multi-data into one message, which would cause a considerable reduction of energy for sending data.

### 3.3. Top-k Query Based on a Tree

**Figure 3:**
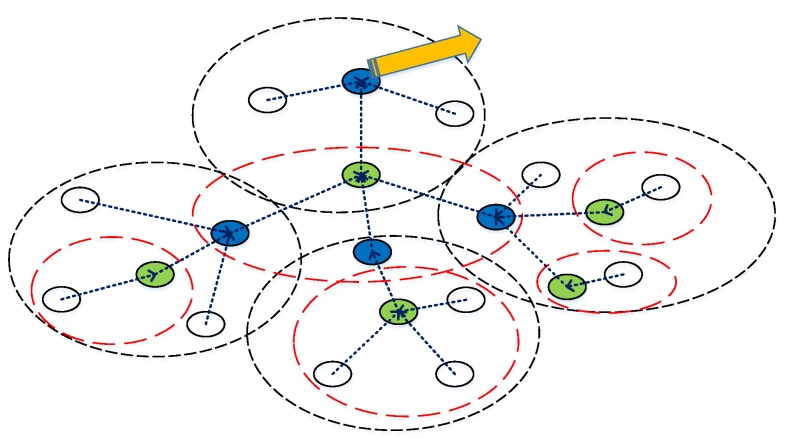
Routing tree in a wireless sensor network.

As shown in Figure 3, it is assumed that all of the sensor nodes in a network communicate with the base station through a routing tree. The construction of routing tree will be discussed in the next section. After the routing tree constructed, each node records its children nodes and the kernel node it connects to. Note that each sensor node must connect to one and only one kernel node. The number of children nodes in a sub-network depends on the topology of the network, and, if a sensor node has no children, it is considered as leaf-node. Correspondingly, a node that has children is kernel node.

Initially, the base station broadcasts a *query message* into the network, and all of the sensor nodes must respond to the base station by their local readings. Every leaf-node, i.e., the border node, embeds its readings into a message and uploads the message to the kernel node it connects to in the routing tree.

The responsibilities of kernel node are slightly more complex than other node. When receiving a message from a child $c_{i}$, first, the kernel node stores the message and then deletes $c_{i}$ from the list of its children. If the list of its children is not empty, the kernel node must store the message in memory and wait until another message is received. If the list is empty, which means all of the children have uploaded their readings, the kernel node begins to aggregate all of the messages that have been received by using LDA. However, if a kernel node waits for a time beyond a preset threshold, it also begins to aggregate the messages. Note that the aggregation process could be conducted along with the arrival of the message. We call this Routing Tree based Local Data Aggregate process LDA-RT.

### 3.4. Routing Tree Construction

To avoid unbalanced energy consumption resulting from fixed route, we introduce a method to construct dynamic route on demand, which is composed of two procedures, i.e., *Interest Forwarding* and *Path Establishment*. The two procedures are both distributed processes that occur at every sensor node. Therefore, they are inseparably intertwined from the view of the entire network.

Assume that each of the sensor nodes in a network has a unique, assigned *ID* that is used to distinguish them from each other. At the beginning of a query task, first, the base station injects an interest message into the network. For the purpose of constructing routing tree, the base station uses limited power to broadcast the interest message. So, only the sensor nodes, that coverage the base station, can receive the interest message. The message is a query or an interrogation, which specifies what a user wants.

The message is a quintuple composed of *qid*, *sid*, *lts*, *rpw* and *k*. Among them, *qid* is the unique identifier of this interest message, which is generated by base station and does not change in the current query round. Term *sid* represents the *ID* of the node that sends this message. At the moment of message generating, base station *ID* is put into *sid*. Term *lts* is the local timestamp of the message sender, and *rpw* the remaining energy, which is useless when the message sender is base station. Term *k* is the number of readings expected to retrieve in this query round.

Consider a sensor node $n_{j}$. When receiving an interest message *M*, $n_{j}$ goes into the *Interest Forwarding* procedure and decides whether to forward the message to its neighbors according to *M.qid*. If a received message is with the *qid* that has been received and stored in cache, the message will not be re-sent. Otherwise, it should be sent to all neighbors of $n_{j}$ after updated, i.e., $n_{j}$.*ID* ⇒ *M.sid*. The rule indicates that a sensor node must forward the interest message only once in a query round.

In addition to disposing of interest message, the sensor node $n_{j}$ begins to run the procedure of *Path Establishment*. In most routing solutions of wireless sensor network, hops between nodes are used as the metric of path length. And meanwhile, the node will be chosen as the next point in the shortest path if the message it forwarded arrives first. This will result in the same path’s being constructed at all times, especially when there are only a few sensor nodes in a network. Assuming that all sensor nodes in a network can keep time synchronization [37], the timestamp of the message can be used as the basis for selecting the next point. It reflects the current load of a sensor node, and a node with a light load should be chosen. But in a practical sense, each sensor node may stay in different status at a given time, i.e., different local time, different time drift, and different amount of remaining energy. All of these will affect the synchronization of time and sending messages. For utilizing the timestamp to balance energy assumption, we use the idea of ‘happened-before’ to select the node that should appear in the routing path.

The relationship of ‘happened-before’ with the set of events of a system (denoted: →) must satisfy three conditions [17]:

(1) If *a* and *b* are events in the same process and a comes before *b*, then *a*→*b*.
(2) If *a* is the event of sending a message by one process and *b* is the event of receiving the same message by another process, then *a*→*b*.
(3) If *a*→*b* and *b*→*c*, then *a*→*c*. Two distinct events *a* and *b* are said to be concurrent events if *a*↛*b* and *b*↛*a*.

Making use of the ‘happened-before’ principle, we propose the following method for constructing the routing path. The basic idea is to choose a node with more residual energy and shorter path to base station as the kernel node. Here the shorter path is not in ‘hop’ sense but in ‘time’ sense, which would carry more information favoring the balance of energy consumption, such as the current load status of nodes.

Consider that node $n_{j}$ has a local time $n_{j}$.*lts* generated by its local clock. When receiving an interest message M, node $n_{j}$ first checks whether a piece of interest message with the same *qid* as *M.qid* has been stored in the interest message cache. If there is no matching interest message in the cache, node $n_{j}$ should write message M to its cache and choose the node denoted by *M.sid* as its kernel node. It also should inform the kernel node after making the choice. Then, it updates its local time $n_{j}$.*lts* by $n_{j}.lts=max\left\{n_{j}.lts,\;M.lts\right\}$. In this case, node $n_{j}$ should forward message *M* with updated fields, i.e., $n_{j}$.*ID* ⇒ *M.sid*, $n_{j}$.*lts* ⇒ *M.lts* and $n_{j}$.*rpw* ⇒ *M.rpw*.

However, if a matching interest message *M*’ exists in the interest cache, the new received interest message *M* will not be broadcast to other sensor nodes. But, the sensor node $n_{j}$ should also compare the message time *M.lts* with *M*’.*lts*. If *M*’.*lts* > *M.lts*, or *M*’.*lts* ≤ *M.lts* and *M*’.*rpw* < *M.rpw*, sensor node $n_{j}$ changes its kernel node to the sensor node with *M.sid* and notes the change to both its previous kernel node and its new kernel. It should also update its local time $n_{j}$.*lts* according to the rule $n_{j}.ts=max\left\{n_{j}.lts,\;M.lts\right\}$.

After an interest message is forwarded to the entire network, a routing tree can be built. Note the following properties associated with the procedure of building routing tree:

- In a query round, each sensor node in the network forwards the interest message only once, no matter how many time it receives the message.
- During the process of constructing routing tree, the kernel node that a sensor node connects to can be updated. But the change is limited to the node itself and does not affect its children nodes.
- Each sensor node forwards interest message immediately after receiving it. This benefits the fast contribution of the routing tree.
- The interest messages that are forwarded by different nodes are allowed to arrive at a sensor node asynchronously. And the ‘happened-before’ mechanism makes it possible to build a dynamic routing path, which is an approximate shortest path and is helpful for accommodating dynamic network topology, balancing the energy consumption and synchronizing the time clocks of the sensor nodes.
- Ideally, after some query rounds, all nodes will have similar local time. And then, remaining energy of nodes will become the deciding factor of routing path.

In general, reconstructing routing tree will consume more energy. But for a sensor network, interest messages should be sent to each node inevitably. Even in filter-based top-k query approach, the configuration about new window size still need to be sent, whenever the measured data exceed the range of the given window. On the other hand, without reconstruction, fixed routing would make some node loss their effectiveness early. The proposed method focuses on build a dynamic routing tree for data collection. And the above properties would reduce the energy consumption of routing tree construction as far as possible.

## 4. Performance Evaluations

### 4.1. Simulation Setup

We implement the proposed LDA-RT algorithms over data-link layer in an NS-3 simulation environment [38] (version 3.21). The implementation covers the details of building routing tree and data aggregation, which are based on the model of media access and physical layers provided for wireless sensor networks in NS-3. For simulating the scene of wearable sensor systems in a larger region, IEEE 802.11b model is employed, which involves YansWifiPhy, YansWifiChannel and AdhocWifiMac. Each sensor node has four modes, i.e., sending a message, receiving a message, idle, and sleeping. Sleeping mode is only used for comparing with other existing approaches. We also implemented a sample filter-based top-*k* query solution with TAG for constructing routing tree, and an improved solution that uses DST.

The energy consumption models we used for the four modes are shown in Equations (3)–(6), respectively,

(3) $$C_{sending}=s*\left(\alpha +\beta *d^{q}\right)$$

(4) $$C_{receiving}=s*\gamma$$

(5) $$C_{idle}=t*\eta$$

(6) $$C_{sleeping}=t*\varepsilon$$

where s is the size of the message, α is a distance-independent parameter, β is a distance-dependent parameter, q is the decay coefficient, d is the distance of the message is transmitted, t is the idle or sleeping time, and γ, ε, and η are constants. As in [5,39,40], we set α=50 nJ/bit, $\beta =100\;\text{pJ}/b/m^{2}$, q=2 in our simulation. In addition, we set d=10 m, γ=50 nJ/bit, η=12.36 mW and ε=0.016 mW. When the distance between two sensor nodes is about 10–14 m, the previous equations can be simplified as shown below:

(7) $$C_{sending}=70*s\;\text{nJ}$$

(8) $$C_{receiving}=50*s\;\text{nJ}$$

(9) $$C_{idle}=12360000*t\;\text{nJ}$$

(10) $$C_{sleeping}=16000*t\;\text{nJ}$$

In order to compare with previous filter-based methods, parts of sensors’ readings are simulated by the database provided by the Live from Earth and Mars project [41] at the University of Washington. We extract 100 similar sub-traces from the temperature traces provided by the project. Each sub-trace contained 800 readings. To reduce the randomness of the results, we repeat the same experiment 10 times and present the average result for each simulation. We use one sub-trace to simulate the readings of sensor nodes. Because of spatial correlation of sensors’ readings, we send successive readings in a sub-trace to neighboring sensor nodes in the simulated network.

In our simulation, for interest message, *qid*, *sid*, *lts*, and *rpw* each take two bytes, and *k* takes one byte. For data message, except for *qid* and *sid*, there are 2 bytes at most for containing the value of readings. Furthermore, in the implementation of filter-based top-*k* query, an interest message contains a 4-byte filter field.

We conduct two kinds of simulations. One is to test the performance of proposed LDA-RT, and the other is to compare LDA-RT with the existing filter-based top-*k* query method. In the former, the initial energy of each sensor node is 1 Joule. In the latter, it is set to 0.01 J for the purpose of comparison.

We simulate multi-hop networks with various numbers of sensor nodes in square and rectangular regions. The base station is placed at the center or at one of the corners, and the distance between two nodes in horizontal direction and in vertical direction is 10 m.

### 4.2. Construction of Routing Tree with Different Parameters

**Figure 4:**
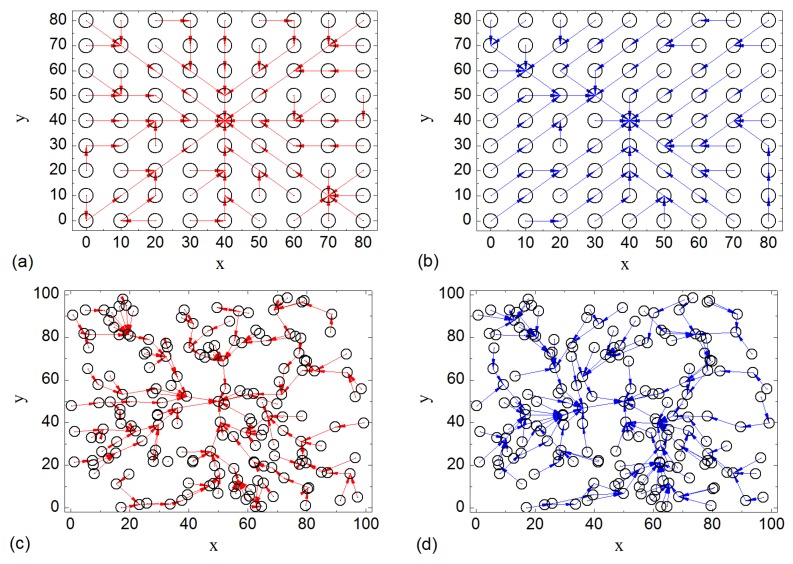
Routing trees constructed by LDA-RT with time drift (−3 ms~+3 ms) and different processing delays: for (**a**) and (**c**), processing delay is 0; for (**b**) and (**d**), the processing delay is 40 ms; (**a**) and (**b**) is the results of grid networks, and (**c**) and (**d**) random networks.

We test the construction process of routing tree using grid network and random network topology. The purpose is to determine whether the routing tree can be established when sensor nodes have time drift in their clock and processing delays. Figure 4 shows part of results. The routing trees that we constructed show that, based on the ‘happened-before’ mechanism, LDA-RT can work well under various conditions. We also note that the routing path constructed by LDA-RT is not but closer to the shortest path in both grid network and random network.

**Figure 5:**
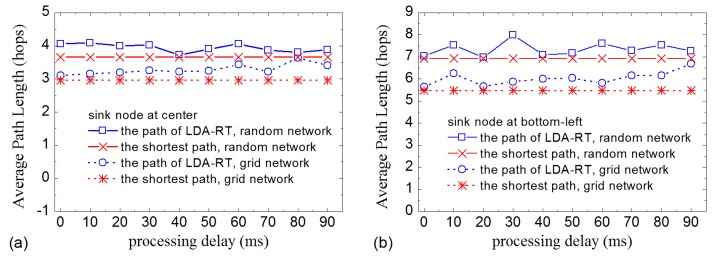
Average path length with different processing delays: (**a**) Base station located at the center; (**b**) Base station located at the bottom-left corner.

**Figure 6:**
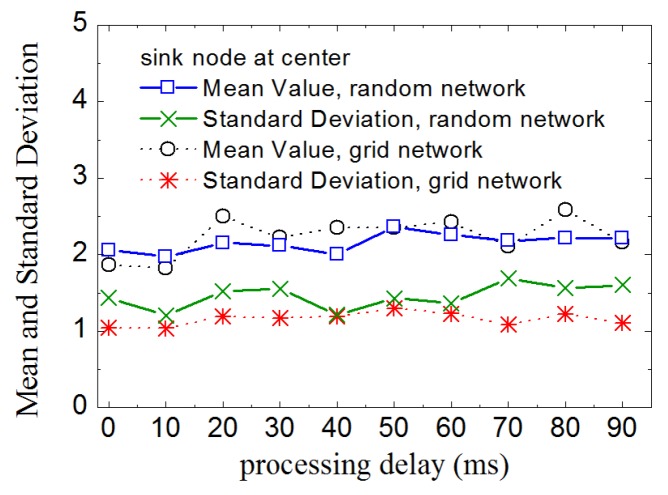
Mean and standard deviation of the number of children nodes of kernel nodes with different processing delays.

**Figure 7:**
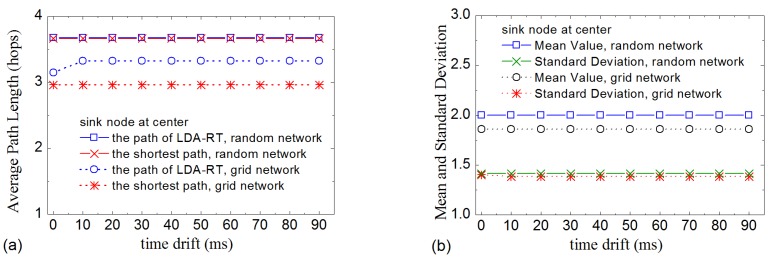
(**a**) Average path length vs. different time drifts; (**b**) Mean and standard deviation of the number of children nodes of kernel nodes with different time drifts.

We further study how the processing delay and time drift affect the routing tree. The method is to measure the path length of the routing tree constructed with different processing delay and time drift. Sensor nodes are assigned a maximum processing delay. Before forwarding an interest message, each node selects a random waiting time from the range of 0 ms to the maximum time, which is used to simulate the processing delay. The results are presented in Figure 5, Figure 6 and Figure 7. Figure 5 shows that the average path length of LDA-RT increases slightly as processing delay increases for grid network. This illustrates that, from a macroscopic perspective, processing delay has no significant influence on the routing tree in LDA-RT. Slightly increasing of average path length would not bring obvious impact on the lifetime of network, comparing with the shortest path. Detailed data indicates that a sensor node has lower probability of being chosen as kernel node when it has higher processing delay. Obviously, this is due to the length of time that passes before interest message is forwarded. Not serving as kernel node means less energy consumption. This is clearly beneficial to the lifetime of nodes that have heavy loads and then encourages the whole network to work longer. Further, LDA-RT will result in non-shortest path. That means these nodes being at the shortest path do not be chosen as kernel node. This is consistent with the results shown in Figure 5, irrespective of whether the base station is located at the center of the network or at one of the corners.

As described in Section 3, kernel nodes in a routing tree conduct data aggregation. So, it is likely to consume more energy than leaf-nodes. In general, the more children nodes that a kernel node has, the more energy it will consume. Even so, when LDA-RT aggregates data, the computations have low complexity and low cost. Therefore, if some kernel nodes have more children nodes than others, they will have an adverse effect on the lifetime of network. Thus, we investigate the number of children nodes of each kernel node in various routing trees with different processing delays. Figure 6 shows the results, in which mean value plots, with values ranging from 1.818 to 2.5 for grid network and from 2.0 to 2.37 for random network, show that processing delay does not have a significant effect on how many children nodes a kernel node has. In addition, under any condition of processing delay, the standard deviation of the number of children nodes is near 1 for grid network and 1.5 for random network, which means that the number of children of every kernel node tended to be close to the mean value. It can also be considered that the energy consumption of each kernel node tends to balance at data aggregation level. Un-uniform distribution of nodes is also the reason that the standard deviation is slightly larger in random network.

Figure 7 gives the results of time drift influence. Because time drift can desynchronize the sensor nodes, it will affect LDA-RT’s construction of the routing tree. The results indicate that, like processing delay, time drift also has an influence on routing tree. However, the influence of time drift is weaker than that of processing delay, as shown in Figure 7a. Numerical analysis indicated that updating local time when receiving an interest message weakens the impact of time drift and helps in constructing a routing tree that is similar to the shortest path. Nevertheless, the impact in random network is larger than that in grid network. The reason is that some nodes in random network have more opportunities to receive interest messages than others. Figure 7b shows the mean and standard deviation of the number of children nodes of kernel nodes for difference time drifts. The curves suggest that time drift has no obvious influence on the distribution of the number of children nodes in LDA-RT for both grid network and random network.

**Figure 8:**
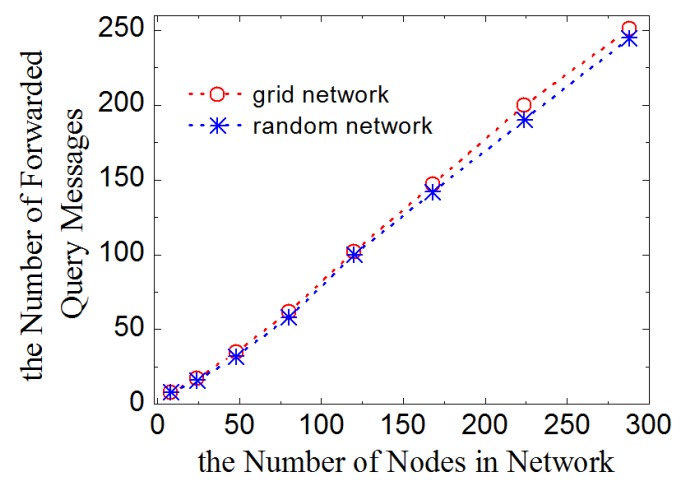
Number of forwarded query message vs. number of nodes.

Another important value is the number of query messages forwarded in one query round in the entire network. Figure 8 shows the results in various sizes of networks excluding messages resent by leaf-nodes. The curve demonstrates that, in LDA-RT, the number of forwarded messages is essentially linear with respect to the number of nodes, which is in agreement with the fact that each node resends the query message only once.

As most studies in literature, we also consider the time that the first node runs out of its energy as the lifetime of network. Therefore, the balance of energy consumption of all nodes is vitally important for the lifetime of network. To inspect the performance of LDA-RT in balancing energy consumption, we run the simulation using a variety of values for parameter *k* and record the final residual energy of nodes on each path in both grid and random networks. We define the final residual energy of a node as the energy at the moment that the first sensor node in network runs out of energy, i.e., its residual energy is insufficient to complete a transmission. For convenience, we set the threshold value to 7 μJ.

**Figure 9:**
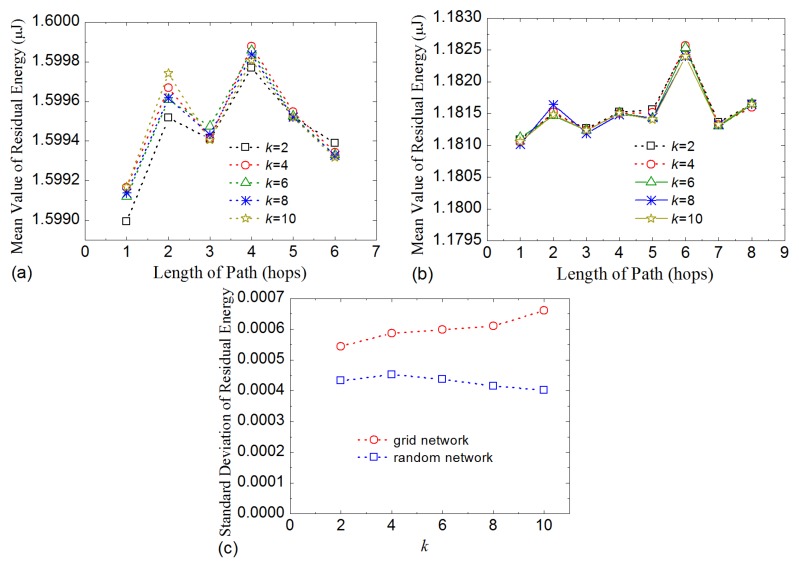
Mean value of residual energy with path length in grid network (**a**) and in random network (**b**); (**c**) Standard deviation of residual energy with different values of k.

Figure 9a shows the mean values of residual energy of nodes in grid networks and Figure 9b in random networks. The results indicate that, when nodes’ paths have the same length, the nodes have approximately the same final residual energy values for different values of parameter *k*. For example, they lie in the range from 1.5995 to 1.5997 μJ for the nodes that have path lengths with the value of 2 in grid network. This indicates that energy consumption is balanced in horizontal direction of the routing tree. However, for a given *k* value, the length of path has no linear effect on the mean value of residual energy. This means energy consumption of nodes is balanced in the direction of routing path under microscope, which is very important for keeping the whole network alive, because the nodes close to base station would usually consume more energy. The fluctuations of values are the result of scale of assembled nodes. The results also indicated that there is no node runs out of energy any earlier than other nodes. Figure 9c shows the standard deviation of residual energy, which further demonstrates that LDA-RT performs well in terms of balancing the energy load. We can notice that random network has smaller standard deviation than grid network. The data details imply that nodes in random network change their kernel node more often, which makes more balanced power consumption.

**Figure 10:**
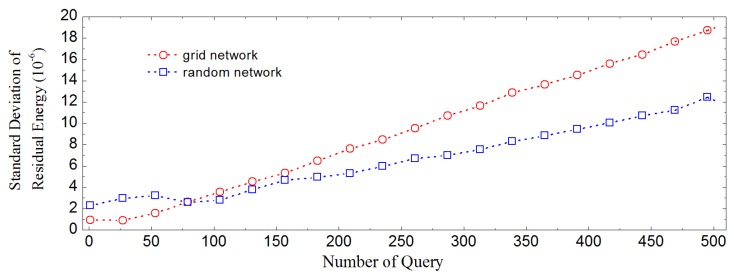
Standard deviation of residual energy vs. the number of query.

**Figure 11:**
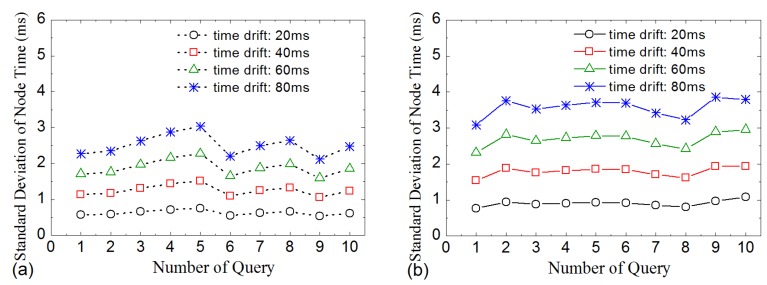
Standard deviation of final time of nodes vs. query number for various time drifts. (**a**) Grid network; (**b**) Random network.

We also invest the relationship between query number and spread extent in the values of residual energy and node time. Figure 10 shows the standard deviation of residual energy at the end time of various numbers of queries. Although the standard deviation increases as the number of queries increases, its value is still small in contrast to the scale of node energy in both grid network and random network. Certainly, there exists difference in the energy expenditure among different nodes. Figure 11a shows that, the main factor affecting the time of a sensor node is clock time drifts, and LDA-RT can help prevent time drift accumulation. For random network, as shown in Figure 11b, the standard deviation of node time is larger than that for grid network. Data show that the time of completing a query is longer in random network than in grid network, because there is more variation of length among different paths in random network.

### 4.3. Comparison of Performance

In this section, we first compare the performance of LDA-RT with original filter-based top-k query approach in terms of energy consumption and network lifetime, which is considered one kind of efficient methods of data aggregation [5]. The method uses TAG, which is a shortest path tree, as its path tree constructor. For the sake of comparability, we employ TAG as the query message path of the original filter-based top-*k* in simulation. Moreover, the query messages of base station are sent to nodes directly by single hop broadcast. We also replace the idle status of sensor nodes to sleep status and set their initial energy to 0.01 J. Because the key factor that affects energy consumption of the original filter-based top-*k* query approach is how many sensor nodes get readings with changed values, which also concerns the power consumption on query message delivery, we simulate the changes by assigning the temperature trace data (mentioned in Section 4.1) to the nodes. Here, “energy consumption” refers to the energy dissipation of all sensor nodes in a given period. And as in [5], the network’s lifetime is defined as the time duration until the first sensor node’s energy is depleted.

**Figure 12:**
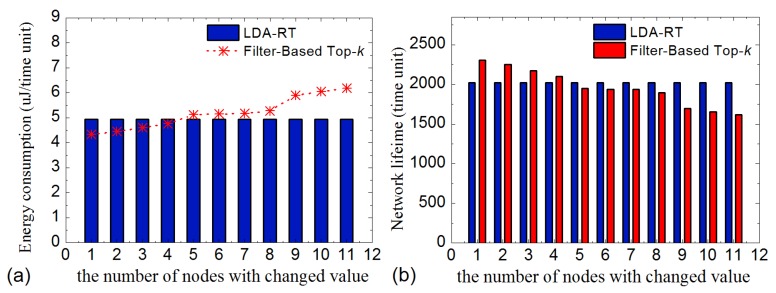
The comparison of (**a**) average energy consumption and (**b**) lifetime between LDA-RT and Filter-Based top-*k*.

Figure 12a shows the simulation results for energy consumption. It is reasonable that average energy consumption of the proposed LDA-RT approach is constant, because energy consumption is not related to the change of reading values in LDA-RT. To the contrary, the energy consumption of filter-based top-k query approach increases as the number of nodes with changed values increases. This can be explained by the fact that increasing the number of changed values causes more frequently updating of windows. This procedure requires more energy on the shortest path tree. In our simulations, when there are four or less changed values, the average energy consumption of filter-based method is improved. However, when five or more values changed in a period, the LDA-RT approach outperformed the filter-based method. The detailed data also indicated that most of energy is consumed during sleeping time rather than by data transmission of. So, if the energy needed to transmit data increases, the curve of filter-based method will increase at a greater rate. It means filter-based method will perform even better than LDA-RT with fewer changes and worse as the number of changed values increases.

Similar to average energy consumption, network’s lifetime is also affected significantly by the number of nodes with changed values in original filter-based method. Figure 12b shows that the network’s lifetime decreases for filter-based method and remains constant for the LDA-RT method as the number of nodes with changed values increases. Detail data indicate that the nodes close to base station consume energy more quickly because they lie on the middle of the fixed path with higher probability.

We next compare the proposed method with an improved filter-based top-*k* method proposed in [3]. The improved method introduced two contributions. One is a filtering-based algorithm, and the other is the authors claimed that the efficiency of top-*k* query algorithms can be improved by choosing a proper underlying logical tree topology. The improved method employs DST (Dominating Set Tree) as message delivery tree.

**Figure 13:**
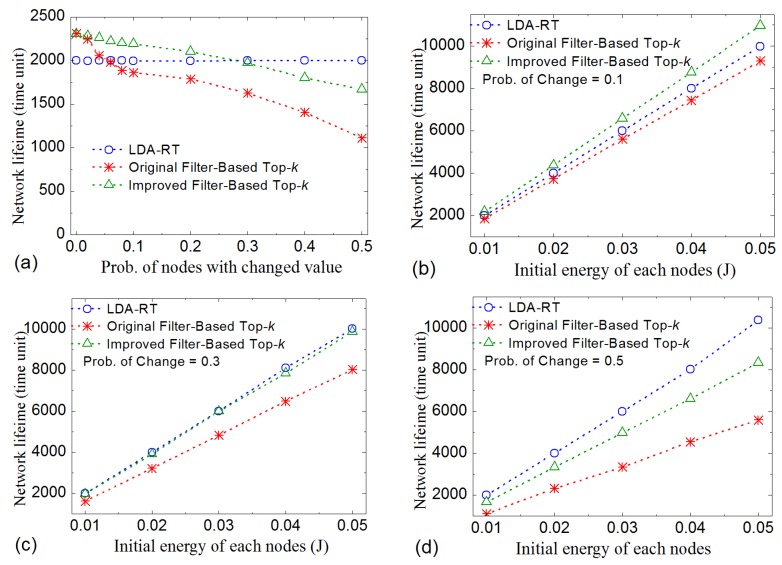
The comparison of lifetime under different conditions. (**a**) Different probability of change; (**b**), (**c**) and (**d**) Different initial energy with probability of change 0.1, 0.3 and 0.5 respectively.

We check the lifetime of network first. In simulation, *k* is set to 10, and the initial energy of each node is 0.01 J. Figure 13a gives the results under different probability of nodes with changed value. When few nodes need to send data, original and improved filter-based top-*k* method have longer lifetime than LDA-RT. With the probability of change increasing, the lifetime of original method drops quickly, and that of improved method drops slowly. When the probability of change is more than 0.3, LDA-RT will result longer lifetime than improved filter-based top-*k* method. Detailed data indicate that two reasons led to low performance of filter-based methods under the condition of high probability of change. One is that message flooding should be employed to generate routing tree in these methods. Fixed routing and the absence of data aggregation are another reason that causes fast energy consumption on some nodes. We also investigate the influence of initial energy of nodes. Results are summarized in Figure 13b–d, which also shows that LDA-RT will have better performance when more nodes need to send their measurements.

Though filter-based methods assume the environment where the values of sensor readings do not change much, they still need to face the issue of unbalanced energy consumption. Furthermore, too strict constraints on the change of reading values will restrict their range of use.

**Figure 14:**
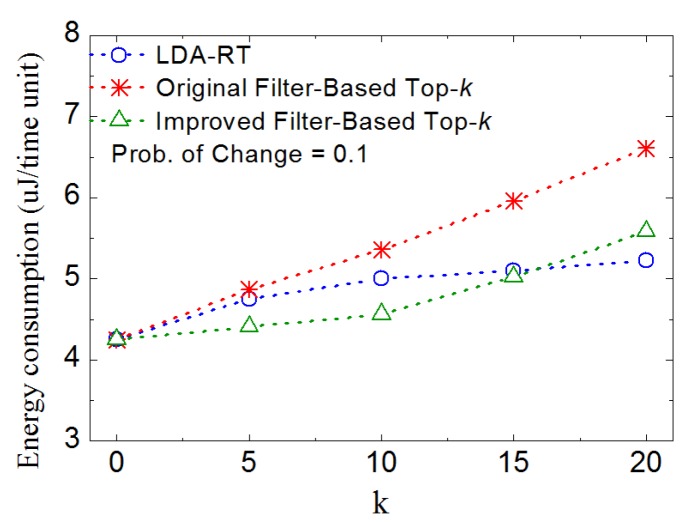
Plots of energy consumption vs. *k*.

We also evaluate the performance under various *k* values. Results are shown in Figure 14. It is observed that, even under the condition of low probability of change, with increasing *k*, the nodes running filter-based top-*k* methods consume more energy. This is because, when more data need to be reported, the probability of updating window size becomes larger. It agrees with the conclusion of [3] that more dynamic of data naturally triggers more updates and nodes-to-root transmissions, which implies that well-designed dynamical routing and data aggregation will show better performance in top-*k* monitoring when data change frequently. These also agree with the researches in ref [5,7,10,15,16,18,19,20].

## 5. Conclusions

In this paper, we studied the issue of top-*k* query in wearable wireless sensor networks and proposed a routing tree-based top-*k* query approach, named LDA-RT. Different from existing works on building routing trees and filter-based query methods, we introduced a ‘happened-before’ rule-based approach to construct the routing tree, which can provide dynamic ability to avoid the weakness of fixed routing. We also presented a distributed local data aggregation algorithm, called LDA. By taking the advantage of routing tree, LDA-RT aggregates measurements locally and sorts them in layers. The proposed aggregation algorithm generates readings with limited sizes and makes the sensor nodes send messages that contain the readings only once in a query round.

We performed a series of simulations to evaluate the performance of the proposed LDA-RT and compared with filter-based top-*k* method. The results indicated the following:

- LDA-RT can build dynamic routing paths quickly, which is helpful for maintaining the balance of energy consumption and synchronizing the time clocks of wearable sensor nodes.
- The method of constructing routing tree also has the ability to round the nodes that have heavy loads, which will reduce the energy consumption of these nodes.
- The energy consumption is balanced in the horizontal direction of routing tree and also in the direction of routing path.
- LDA-RT outperforms filter-based method on energy consumption and network’s lifetime when the readings of sensor nodes changes frequently, especially when there is a heavy load of data to be transmitted.

Although the proposed LDA-RT shows better performance in many aspects, it should still be improved in reduction of energy consumption by query message and data transmission. Meanwhile, the adaptability to dynamic topology and changing data in large range must be preserved. It could be a breakthrough but must be difficult to assemble the advantages of different methods to meet these needs. In our future work, we plan to include filter based top-*k* query methods as an extension of LDA-RT to decrease the cost of transmitting data even further.
